# Developing and implementing an interventional bundle to reduce mortality from gastroschisis in low-resource settings

**DOI:** 10.12688/wellcomeopenres.15113.1

**Published:** 2019-03-08

**Authors:** Naomi Wright, Francis Abantanga, Michael Amoah, William Appeadu-Mensah, Zaitun Bokhary, Bruce Bvulani, Justine Davies, Sam Miti, Bip Nandi, Boateng Nimako, Dan Poenaru, Stephen Tabiri, Abiboye Yifieyeh, Niyi Ade-Ajayi, Nick Sevdalis, Andy Leather

**Affiliations:** 1 King's Centre for Global Health and Health Partnerships, School of Population Health and Environmental Sciences, King's College London, London, SE5 9RJ, UK; 2 Department of Surgery, Tamale Teaching Hospital, Tamale, P.O. Box TL 16, Ghana; 3 Department of Surgery, Komfo Anokye Teaching Hospital, Kumasi, P.O.Box 1934, Ghana; 4 Department of Paediatric Surgery, Korle-Bu Teaching Hospital, Accra, P.O. Box 77, Ghana; 5 Department of Paediatric Surgery, Muhimbili National Hospital, Dar es Salaam, P.O Box 65000, Tanzania; 6 Department of Paediatric Surgery, University Teaching Hospital of Lusaka, Lusaka, 10101, Zambia; 7 Global Health and Education Department, University of Birmingham, Birmingham, B15 2TT, UK; 8 Department of Paediatrics, Arthur Davison Children's Hospital, Ndola, Zambia; 9 Department of Paediatric Surgery, Kamuzu Central Hospital, Lilongwe, P.O. Box 149, Malawi; 10 McGill University, Montreal, Quebec, H3A 0G4, Canada; 11 Department of Paediatric Surgery, King's College Hospital, Denmark Hill, London, SE5 9RS, UK; 12 Centre for Implementation Science, King's College London, London, SE5 8AF, UK

**Keywords:** Gastroschisis, Intervention, Mortality, Low-Resource Setting, LMIC, Implementation, Congenital Anomaly, Neonatal Surgical Care

## Abstract

**Background: **Gastroschisis is associated with less than 4% mortality in high-income countries and over 90% mortality in many tertiary paediatric surgery centres across sub-Saharan Africa (SSA). The aim of this trial is to develop, implement and prospectively evaluate an interventional bundle to reduce mortality from gastroschisis in seven tertiary paediatric surgery centres across SSA.

**Methods:** A hybrid type-2 effectiveness-implementation, pre-post study design will be utilised. Using current literature an evidence-based, low-technology interventional bundle has been developed. A systematic review, qualitative study and Delphi process will provide further evidence to optimise the interventional bundle and implementation strategy. The interventional bundle has core components, which will remain consistent across all sites, and adaptable components, which will be determined through in-country co-development meetings. Pre- and post-intervention data will be collected on clinical, service delivery and implementation outcomes for 2-years at each site. The primary clinical outcome will be all-cause, in-hospital mortality. Secondary outcomes include the occurrence of a major complication, length of hospital stay and time to full enteral feeds. Service delivery outcomes include time to hospital and primary intervention, and adherence to the pre-hospital and in-hospital protocols.  Implementation outcomes are acceptability, adoption, appropriateness, feasibility, fidelity, coverage, cost and sustainability.

Pre- and post-intervention clinical outcomes will be compared using Chi-squared analysis, unpaired t-test and/or Mann-Whitney *U *test. Time-series analysis will be undertaken using Statistical Process Control to identify significant trends and shifts in outcome overtime. Multivariate logistic regression analysis will be used to identify clinical and implementation factors affecting outcome with adjustment for confounders.

**Outcome: **This will be the first multi-centre interventional study to our knowledge aimed at reducing mortality from gastroschisis in low-resource settings. If successful, detailed evaluation of both the clinical and implementation components of the study will allow sustainability in the study sites and further scale-up.

**Registration: **ClinicalTrials.gov Identifier NCT03724214.

## Introduction

### The problem

Gastroschisis, one of the commonest congenital anomalies, exhibits great disparity in outcome globally ^1, 2^. Over the last half century, mortality in HICs has fallen from over 90% in the 1960s to less than 4% today, with the majority of survivors proceeding to live a full, normal life ^3– 5^. Such improvements have not been realised in low- and middle-income countries (LMICs) where the majority of births, and hence cases, occur. Mortality has been reported as: 98–100% Uganda, 100% Cote d’Ivoire, 84% Zimbabwe, 80% Iran, 79% Jamaica, 75% Nigeria, 60% Malawi, 34% Turkey, 29–65% South Africa, and 23–57% China ^1, 2, 6– 13^. Some middle-income countries have managed to reduce mortality from gastroschisis in recent decades, such as Thailand from 25% in 1986 to 8% in 2009 ^14, 15^.

The incidence of gastroschisis continues to increase globally ^3, 14, 16– 29^. In LMICs, not only is the true incidence increasing, but also the number of patients presenting to a healthcare facility; in Pretoria, South Africa there has been a 35-fold increase in cases between 1981 to 2001 ^17, 30^. The aetiology of gastroschisis is unknown ^3^. Young maternal age (<20 years) has been identified as the strongest risk factor ^3, 16, 29, 31– 33^. Other associations include low body mass index, smoking, use of anti-depressants, exposure to contraceptive hormones during the first trimester, pre-gestational or gestational diabetes, alcohol, cocaine and methamphetamine, although findings are inconsistent ^3, 16, 27, 32, 34– 43^. There has been little investigation into causation in LMICs and indeed in a Ugandan study, the majority of mothers were between 20 and 29 years of age despite a high proportion of teenage pregnancies compared to HICs and they denied smoking or taking drugs ^6, 44^.

An estimated 10–15% of neonates with gastroschisis have an extra-intestinal congenital anomaly (cardiac, genito-urological, musculoskeletal and neurological); these findings are consistent in studies across the globe including both HICs and LMICs ^3, 9, 45– 49^. However, the proportion with intestinal pathology or ‘complex gastroschisis’ is greater in LMICs; up to 25% compared to 10% in HICs (defined as intestinal ischaemia, necrosis, perforation or atresia) ^7, 10^. A lack of antenatal diagnosis, delivery outside a tertiary paediatric surgery centre, and inadequate pre-hospital management and transfer results in significant delays in reaching care, during which time the bowel is exposed, contaminated, damaged and/ or torted on the vascular pedicle resulting in postnatal ischaemia and necrosis ^1, 6, 7, 12^. In addition, neonates commonly arrive hypovolaemic, hypothermic and septic ^6, 7, 11, 44^. In HICs, complex gastroschisis is associated with a significantly higher mortality at 17% compared to 2% for simple gastroschisis ^50^. In LMICs, the additional systemic compromise results in the majority of neonates with gastroschisis (both simple and complex) dying within a median of 4-days at a healthcare facility ^6, 7, 44^.

### Current practice and guidelines

There are a number of components to successful gastroschisis management including: antenatal diagnosis, delivery in a tertiary paediatric surgery centre or adequate pre-hospital management and transfer, pre-intervention resuscitation, bowel reduction and defect closure, and post-intervention neonatal care including provision of parenteral nutrition (PN) until enteral feeding is established. In HICs, practically all neonates are diagnosed antenatally and a delivery plan is constructed resulting in either delivery at a tertiary paediatric surgery centre or rapid stabilisation and transfer to such a unit ^51^. Even in HICs, the latter has been shown to result in poorer outcomes ^52^. Methods of bowel reduction and defect closure vary widely ^53^. The two most commonly utilised techniques are primary closure in an operating room (OR) or cotside application of a preformed silo (PFS) with serial reductions over several days followed by cotside sutureless closure or closure in the OR ^54– 56^. Neonates are managed in an intensive care unit (ICU) by a multi-disciplinary team (MDT) of neonatologists, paediatric surgeons, anaesthetists, specialist nurses, and sometimes paediatric gastroenterologists. Ventilation is available if required and all neonates receive central intravenous (IV) access and PN. For neonates with simple gastroschisis the median duration of PN is 23-days and length of hospital stay (LOS) 36-days ^4^. Earlier time to first enteral feed has been associated with a shorter duration of PN and LOS in both HIC and LMIC settings ^31, 57, 58^.

In LMICs, many women do receive antenatal care as per World Health Organisation (WHO) guidelines; however, these do not include an ultrasound scan, which is required to detect congenital anomalies ^59– 64^. In a prospective cohort study of 42 neonates with gastroschisis in Uganda, 24% of mothers underwent a second trimester ultrasound by a technician holding a diploma ^6^. However, just one had a correct antenatal diagnosis and therefore over 95% were born outside of a tertiary paediatric surgery centre ^6– 8^. In this study, 81% were born in a first or second level healthcare facility, but appropriate care was not instigated; 81% were without appropriate bowel coverage, 54% without IV access, 83% without an nasogastric (NG) tube, 52% were breastfeeding, and only 58% arrived within 12-hours of delivery ^6^. Only 35% travelled by ambulance ^6^. In an international survey on gastroschisis, primary closure rates were reported as similar in LICs to HICs; however, the majority of staged closure was noted to be undertaken by make-shift surgical silos sutured to the abdominal wall rather than spring-loaded PFSs ^1^. In this survey, only 36% of low-income country paediatric surgery centres had a neonatal ICU and only 19% had access to PN ^1^. It is estimated that a full term, healthy neonate may be able to survive for 1-month without nutrition ^65^.

In HICs, over the last decade, there has been a trend towards routine utilisation of the PFS, applied at the cotside with subsequent cotside closure of the defect ^54^. A randomised controlled trial (RCT) comparing primary closure with PFS reported a lower requirement for ventilation in the PFS group, but no difference in the duration of PN, LOS, sepsis or necrotising enterocolitis (NEC) ^55^. A meta-analysis comparing primary closure with staged closure reported fewer ventilation days (p<0.0001), reduced time to first feed (p=0.04) and lower infection rates (p=0.03) in the latter group amongst studies with least selection bias ^56^. A subsequent systematic review and meta-analysis comparing PFSs with all alternative strategies reported that routine use of PFSs reduced ventilatory requirements with no difference in other outcomes ^54^. Indeed, many neonates in the PFS group required no ventilatory support ^54^. Allotey *et al*. reported lower mean airway pressures and inspired oxygen requirement, higher urine output and no inotropic support in neonates managed with a PFS compared to primary closure of which 43% required inotropes; this reflects the reduced risks of abdominal compartment syndrome (ACS) with PFS use ^66^. PFSs have the added benefit that they can be applied by a suitably trained registrar day or night at the cotside, negating the need for an emergency out-of-hours operation by a consultant ^54^.

Use of the PFS has potentially much greater benefits in the low-resource setting. It permits a focus on resuscitation and neonatal care during the first few days in hospital when the neonate is critically unwell, whilst avoiding neonatal anaesthesia and surgery altogether. This is important because these neonates typically have a higher American Association of Anaesthesiologists (ASA) score at the time of intervention compared to HICs counterparts, which is associated with greater peri-operative mortality ^7^. In addition, neonatal anaesthesia holds greater risks in LMICs due to lack of training and resources, and OR availability in LMICs is often limited and commonly prioritised to other children and adults who are deemed to have a greater chance of survival ^6, 7^. PFSs reduce the risk of ACS associated with primary closure; the latter is an even greater risk in LMICs because the exposed bowel typically becomes oedematous, matted and covered in peel ^54^. Reduced ACS in turn reduces requirements for neonatal ICU resources which are limited in such a setting ^15, 66^. However, PFS have not been widely adopted in LMICs due to unavailability, cost and lack of appropriate training ^1^. More recently, much cheaper alternatives such as the Alexis wound retractor have resulted in significantly improved survival in countries such as Japan, Malaysia and Mexico ^67– 71^. Other techniques such as flap closure, umbilical turban and the Bianchi technique have been used with some success ^72– 75^.

Most HIC paediatric surgery centres have a protocol for the management of neonates with gastroschisis. There is evidence that implementation of protocols can improve care and outcomes in critically ill paediatric patients ^76^. Protocols are not commonly utilised in LMICs at present; however, the inclusion of a standardised care protocol will be important for any quality improvement project. Recently, global guidelines for the management of neonates and children requiring surgery in LMICs have been produced, including recommendations for gastroschisis care ^77, 78^. It is recommended that all primary and secondary level healthcare facilities are able to safely and effectively resuscitate and stabilise a neonate with gastroschisis and transfer to a tertiary paediatric surgery centre for definitive management.

### Quality improvement projects focussed on neonatal care in LMICs

Previously published projects aimed at improving outcomes for both surgical and medical neonates in LMICs provide a vital insight and evidence to assist in the development of the interventional bundle and implementation strategy (defined as methods or techniques used to enhance the adoption, implementation, and sustainability of a clinical program or practice). To date, there has been just one published quality improvement (QI) project aimed at improving outcomes for neonates with gastroschisis in LMICs to our knowledge ^71^. There has been one QI project focussed on reducing LOS for neonates with gastroschisis in a HIC, two studies focussed on improving outcomes for neonates requiring surgery in LMICs and numerous studies on improving general neonatal outcomes in low-resource settings ^79– 105^.

In Mexico, Zalles-Vidal *et al*. implemented a QI protocol consisting of referral/transport advice, primary cotside closure or staged reduction at the cotside with an Alexis Wound Retractor without general anaesthesia, PICC lines, and early feeding; mortality was reduced from 22% to 2% ^71^. They reported a reduction in need for ventilation from 100% to 57%, a reduction in mean ventilator days from 14 to 3, a reduction in TPN days from 27 to 21 and a reduction in sepsis from 70 to 37% ^71^. Mansfield *et al*. managed to reduce the median LOS for neonates with uncomplicated gastroschisis in the US from 34 to 29 days with a QI project utilising an evidence-based protocol implemented with repeated Plan, Do, Study, Act (PDSA) cycles ^80^. Their implementation strategy included MDT educational sessions with nurses, neonatal staff, surgical staff, anaesthetists and gastroenterologists with quarterly updates and amendments to the protocol as required ^106^. All members of the MDT were involved in the original development of the protocol. Using time-series analysis, they observed that the reduced LOS corresponded with a change in surgeon practice to bedside silo placement. Their future recommendations include preferential bedside PFS placement, sedation protocol to minimise need for ventilation, feeding advancement regime and improved parental/infant skin-to-skin contact.

Ekenze *et al*. undertook a QI project focussed on co-ordinating interdisciplinary collaboration to improve neonatal surgery outcomes in Nigeria ^81^. The intervention involved short-term neonatal surgery training for the paediatric surgeons, nurses and anaesthetists in Europe and the US followed by MDT training locally upon their return. This resulted in a significant reduction in overall mortality from 48.9 to 22.7% (p<0.05) and a non-significant reduction in complications from 55.3 to 38.6% (p>0.05). The commonest causes of death were sepsis, anaesthesia and respiratory compromise. Common challenges they faced included delayed presentation, inadequate facilities and a defective health insurance scheme. They recommended foreign trainers to visit and train local specialists in peri-operative nursing care and infection control including protocols for cleaning cots/ incubators and minimising thoroughfare in newborn wards.

Khan *et al*. reported their experience with joining an international QI collaboration on congenital heart surgery in Pakistan ^79^. Their intervention focussed on reducing surgical site infection (SSI) through MDT education and webinars, hand sanitisers installed in ICUs and patients’ bedsides, targets for hand hygiene displayed prominently on notice boards, protective clothing during procedures and separation of the adult and paediatric ICU bays with traffic control. Pivotal to their implementation strategy was nurse empowerment and hospital management buy-in. A senior nurse was put in charge of the project, acting as the liaison with hospital management and the training lead for nursing. Nurse training included active participation on ward rounds, assertive communication and clinical training. This uses Kanter’s theory that empowering nurses through resources, support and opportunities results in more accountability for their work, more commitment to the organisation, higher job satisfaction and retention rates. Their project resulted in a significant reduction in SSI and bacterial sepsis rate from 30 to 1% (p=0.0001) and a non-significant reduction in mortality from 9 to 6% (p=0.17).

There are a number of QI projects focussed on improving neonatal care and outcomes in LMICs including 25 before and after studies, 2 non-randomised interventional studies and one RCT ^82– 105^. The majority of these are meso level, single-centre, educational interventions. Others include service re-organisation (increased access of mothers to neonates and triage systems) and reference materials (protocol implementation) ^84, 92, 97, 98^. Promotors of success included the presence of a local champion and motivated key individual, particularly nursing supervisor ^83, 87, 100^. One study mentioned the value of support from the Ghanaian Health Service ^83^. Barriers to success included: over-burdened staff, insufficient equipment and government policies enforcing re-distribution of staff away from study sites ^85, 87, 102^. The majority of successful initiatives involved a multi-faceted approach including protocol development and implementation, MDT training and education, nurse empowerment and a greater involvement of mothers in care-giving ^84, 86, 98^.

Examples include Agarwal *et al*. who implemented a bundle of simple interventions to improve neonatal mortality in Pakistan ^84^. This included: training and utilising mothers as caregivers, aggressive enteral feeding, infection control measures, protocol-based management with abandonment of unnecessary interventions, rational use of antibiotics and training/empowering nurses. This resulted in a significant reduction in overall mortality from 29.3/1000 to 20.3/1000. Bastani *et al*. undertook a RCT in Iran comparing routine neonatal care practice to ‘family centred care’ including maternal education, presence at the cotside and involvement in monitoring and care ^97^. This resulted in a significant reduction in LOS and neonatal readmission and higher parental satisfaction scores. Similarly, Bhutte *et al*. in Pakistan showed a QI project focussed on maternal training and empowerment reduced LOS for very low birth weight neonates from 34 to 16 days without increasing complication rates or readmission ^98^. The maternal training included: regular monitoring of vital signs, administering breastmilk via a NG tube, handwashing, minimising other family visitors, co-bedding mother and infant, and awareness training regarding danger signs and when to seek help.

These studies highlight the need to focus on sepsis, anaesthesia and respiratory compromise as leading causes of death. They emphasise common challenges in low-resource settings including delayed presentation, overburdened staff, re-distribution of staff, inadequate facilities and defective health insurance schemes. Finally they offer some guidance to potential solutions: preferential bedside PFS placement, use of a sedation protocol to minimise need for ventilation, use of a feeding advancement regime, improved maternal/infant contact, maternal involvement in care, nurse empowerment, hospital management buy-in, a local champion, education/training particularly with regards to nursing care and infection control, reduction in ward thoroughfare, resource provision and protocol implementation. Importantly, the use of a multi-faceated approach is underscored.

### Implementation science

Implementation science is the study of methods to promote the adoption and integration of evidence-based practices, interventions and policies into routine health care and public health settings. This includes the use of evidence-based implementation strategies and theory in the project or study design and evaluation of the implementation outcomes in addition to clinical outcomes. There are eight defined implementation outcomes, as follows ^107^:

•   Acceptability: Perception amongst stakeholders that the new intervention is agreeable.

•   Adoption: Intention to apply new intervention.

•   Appropriateness: Perceived relevance of the intervention for the setting and problem.

•   Feasibility: Extent to which an intervention can be applied.

•   Fidelity: The proportion of management protocol components completed as intended.

•   Coverage: The proportion of eligible patients who actually receive the intervention.

•   Cost: Costs of the intervention, including the delivery strategy.

•   Sustainability: Extent to which a new intervention becomes routinely available/ is maintained post-introduction.

Although the above QI projects commonly describe the intervention and clinical outcomes, none have formally evaluated implementation outcomes. Such information is vital to understand which components of both the interventional bundle and the implementation strategy are effective or not. For example, if an intervention is not successful is it because the intervention itself is ineffective or is it because it was not effectively implemented and hence not used in practice. In this study, implementation science techniques will be utilised to optimise the study design, implementation of the interventional bundle and evaluation of the outcomes to optimise its success and reproducibility ^108^.

## Protocol

### Research question

Can implementation of an evidence-based interventional bundle reduce mortality from gastroschisis in low-resource settings?

## Aim

To develop, implement and prospectively evaluate an interventional bundle to reduce mortality from gastroschisis in seven tertiary paediatric surgery centres across sub-Saharan Africa.

## Objectives

1)   To undertake a systematic review of interventions used to reduce mortality from gastroschisis in LMICs.

2)   To undertake a qualitative analysis of centres in LMICs with lower and higher gastroschisis-related mortality to identify successful healthcare initiatives and potential barriers and facilitators for improved outcomes.

3)   To undertake a Delphi process, using the above results and experts in the field, to determine an interventional bundle aimed at improving survival of neonates born with gastroschisis in low-resource settings.

4)   To implement and prospectively evaluate the interventional bundle at seven tertiary paediatric surgery centres across SSA aimed at reducing the mortality in neonates born with gastroschisis.

## Methods

### Development of the interventional bundle

Utilising a detailed literature search and expertise within the study team an interventional bundle was drafted, study funding was gained and ethical approval achieved at all study sites. Further research is being undertaken including a systematic review, qualitative study and Delphi process to provide additional evidence and to optimise the implementation strategy. The interventional bundle will be further defined and modified if new evidence is identified and the ethical approval amended if required. Study leads and team members from all seven sites have been and will continue to be involved in the development of the interventional bundle throughout the process to optimise the study design and to ensure effective implementation of the intervention.

***Systematic review.*** The systematic review will evaluate all published and unpublished literature regarding interventions to reduce mortality from gastroschisis in LMICs ^109^. Articles on a wider range of gastro-intestinal congenital anomalies will also be included to evaluate generic neonatal surgery interventions utilised in LMICs, which may also benefit neonates with gastroschisis.

***Qualitative study.*** The qualitative study will involve semi-structured interviews of both neonatal surgical care providers achieving lower mortality from gastroschisis in LMICs to identify successful healthcare initiatives and implementation strategies, and those with high mortality rates (at the study centres) to identify specific barriers, ineffective practices and potential solutions. The ultimate aim of this part of the study is to identify context-appropriate, implementable and scalable interventions. It is also an important step for building rapport and gaining study input and buy-in from different members of the MDT at the study sites. A protocol for this study is forthcoming.

***Delphi process.*** Interventions to be considered during the Delphi process will be identified from the literature review, systematic review and qualitative study. An online, phased Delphi process will be developed. In round one, experts will be asked to score interventions on pre-determined Likert type scales based on how important and feasible they are for achieving a reduction in mortality from gastroschisis in a low-resource environment. During rounds two, and three if necessary, experts will be presented with the same list of interventions and a graphical representation of the scores for each intervention during round one and round two, respectively. They will be asked to re-score each intervention taking into account how important and feasible other members of the expert panel felt it to be. If required, a consensus meeting will be undertaken via online teleconference, involving a variety of MDT members from each of the study centres to finalise the core and adaptable components of the interventional bundle to be implemented. A protocol for this study is forthcoming.

***In-country co-development meeting.*** During the implementation process, in-country co-development meetings will be undertaken with all members of the MDT caring for neonates with gastroschisis at each institution to discuss and decide upon the adaptable components of the interventional bundle and to approve an appropriate implementation strategy to suit the local teams at each site.

### Outline of the interventional bundle

The interventional bundle will consist of both pre-hospital and in-hospital components. Each of these will have core components that will be consistent across the seven study centres and adaptable components for optimisation to the local context ( Table 1). The content and detail of the core and adaptable components will be further defined utilising the results of the systematic review, qualitative study and Delphi process. A summary of the drivers to achieving a reduction in gastroschisis mortality is summarised in Supplementary File 1 ^110^.

**Table 1.** An outline of the core and adaptable components of the interventional bundle.

Pre-Hospital	In-Hospital
Core	
A pre-hospital management protocol will be implemented at first and second level healthcare facilities (both government and private) that refer patients with gastroschisis to the study centres. These will be identified by the study leads at each site. The protocol will include: • Covering the bowel in clear plastic • Administering intravenous (IV) fluids • Keeping the neonate warm • Transferring to the study centre as soon as possible	• Use of a standardised protocol for care. • Neonatal resuscitation and ward care including IV access, IV fluids, maintenance of normothermia, appropriate antibiotics, regular monitoring and infection control. • Gastroschisis reduction and sutureless closure using a preformed silo, or equivalent, and avoidance of neonatal anaesthesia and surgery. • Early establishment of breastfeeding and enhanced enteral feeding programme.
Adaptable	
The method of dissemination and implementation of the pre-hospital management protocol will vary at each centre according to the optimal strategy as determined by the local team. Strategies include: • Wide dissemination of a guideline, leaflet and poster detailing the protocol with pictorial representations of the steps involved via post +/- back-up with telephone communications. • In addition to the above, site visits to selected referral centres for education and training to enhance implementation of the protocol. Visits and training will be undertaken by local MDT members and the principal investigator. • As an alternative to visiting referral hospitals, a gastroschisis study day at the study centre with MDT members from referral hospitals invited for education and training to enhance implementation of the protocol. • Dissemination of the protocol through existing government health policy pathways. • Radio and/or television campaign.	• Administration of a short period of parenteral nutrition for neonates who have survived to 7-days of life. • Maternal involvement in monitoring and basic management. • Management of neonates with gastroschisis on the neonatal ICU if available.

### Implementation of the interventional bundle

***Study design.*** A hybrid type 2 effectiveness-implementation pre-post study design will be utilised, which focusses equally on the effectiveness of the intervention through analysing clinical outcomes and effectiveness of the implementation through analysis of the service delivery and implementation outcomes ^111^.

The theoretical underpinning of the intervention and implementation strategy is based on the normalisation process theory (NPT) ^112^. This is a theory developed by implementation researchers in order to provide explanation and understanding of how a novel intervention becomes standard practice within a healthcare setting ^113– 115^. Table 2 highlights the four constructs of the NPT and how they are addressed in the study design. The associated validated survey, NoMAD, will be utilised to evaluate MDT members opinions on each of these four areas at the end of the MDT training day (detailed below) and deficient areas will be actively targeted for improvement during the 4-week in-country implementation phase ( Supplementary File 2) ^110, 116^. The survey will be repeated at the end of the study to assess if and how perceptions have changed between implementation and study completion.

**Table 2.** Normalisation process theory constructs used in the study design.

Normalisation Process Theory Constructs	How these will be addressed in the study
**Coherence:** • Participants distinguish the intervention from current ways of working • Participants collectively agree about the purpose of the intervention • Participants individually understand what the intervention requires of them • Participants construct potential value of the intervention for their work	• Involvement of all key MDT members in the development of the study and interventional bundle to be implemented • MDT simulation training (detailed below) • Through raising awareness about the possibility of improved outcomes using examples from other LMIC settings (through sharing the results of the systematic review and qualitative study with MDT members and discussion during the Delphi process consensus meeting, in-country co-development meeting and MDT training day). A leaflet will be developed and distributed amongst MDT members detailing successful gastroschisis management initiatives in other LMIC settings.
**Cognitive participation:** • Key individuals drive the intervention forward • Participants agree that the intervention should be part of their work • Participants buy-in to the intervention • Participants continue to support the intervention	• Study leads have requested participation in the study from each site • Some additional team members have already been identified with an interest to participate in the study • Identification of key individuals from the MDT to participate in the qualitative study and Delphi process • In-country co-development meeting to ensure local acceptability and feasibility and to provide local ownership
**Collective action:** • Participants perform the tasks required by the intervention • Participants maintain their trust in each other’s work and expertise through the intervention • The work of the intervention is appropriately allocated to participants • The intervention is adequately supported by its host organisation	• Locally determined roles and responsibilities within the study • Fidelity will be monitored and problems adhering to the protocol discussed and troubleshooted at the monthly MDT meetings • MDT simulation training to practice and trouble shoot in the simulated setting prior to implementation in practice • Inclusion of the hospital management in the development process, in-country co- development meeting and monthly MDT meetings
**Reflexive Monitoring:** • Participants access information about the effects of the intervention • Participants collectively assess the intervention as worthwhile • Participants individually assess the intervention as worthwhile • Participants modify their work in response to their appraisal of the intervention	• Real-time study outcomes and fidelity will be fed back to the MDT on a monthly basis and any problems with implementing components of the protocol will be discussed and troubleshooted • Challenges and successes from other study teams will also be shared so that teams can learn from each other’s experience

The Expert Recommendations for Implementing Change (ERIC) compilation of implementation strategies was utilised in the study design; 48 of the 73 strategies have been incorporated ^117^. The majority of chosen strategies rank as both important and feasible in the concept mapping study by Waltz *et al*. ^118^. The following guidelines have been complied with to optimise the study design, evaluation and planned reporting: Standard Protocol Items: Recommendations for Interventional Trial (SPIRIT); Medical Research Council guidance on developing and evaluating complex interventions; Standards for reporting implementation studies (StaRI); Template for Intervention Description and Replication (TIDieR); and Standards for Quality Improvement Reporting Excellence (SQUIRE) ^119– 125^.

***Study sites.***

The study sites include seven tertiary paediatric surgery centres as follows:

1) Korle-Bu Teaching Hospital, Accra, Ghana
2) Tamale Teaching Hospital, Tamale, Ghana
3) Komfo Anokye Teaching Hospital, Kumasi, Ghana
4) University Teaching Hospital, Lusaka, Zambia
5) Arthur Davison Children’s Hospital, Ndola, Zambia
6) Kamuzu Central Hospital, Lilongwe, Malawi
7) Muhimbili National Hospital, Dar es Salaam, Tanzania

The study sites were included based on the following criteria:

- Presence of a local champion/ study lead who requested/ agreed to participate in the study
- Tertiary Paediatric Surgery Centre where patients with gastroschisis are managed
- At least 1–4 cases of gastroschisis per month
- Above 90–95% mortality from gastroschisis at baseline

***Stakeholders.*** Stakeholders at micro (patient/ parents), meso (healthcare facility teams) and macro (regional/ national) levels will be incorporated, with the main interventions being at the micro-meso level. The key stakeholders are summarised in Table 3. All members of the MDT caring for neonates with gastroschisis are included as stakeholders. The study leads from each centre have identified a lead nurse and neonatologist and/or paediatrician and have held local team meetings to discuss and contribute to the study design and protocol. The study leads, lead nurse and lead neonatologist and/or paediatrician will be invited to participate in the qualitative study and Delphi process. The principal investigator (PI) will meet in-person with the study lead, lead nurse, lead neonatologist and/ or paediatrician, hospital management and the wider MDT involved in caring for neonates with gastroschisis for a co-development meeting when in-country. This will finalise the adaptable components for the interventional bundle. All in-hospital stakeholders will be involved in the implementation of the interventional bundle and follow-up MDT meetings to monitor progress and troubleshoot.

**Table 3.** Key stakeholders for the pre-hospital and in-hospital components.

Stakeholders for pre-hospital component	Stakeholders for in-hospital component
Regional and national health boards Hospital management Nurses, doctors, surgeons and allied health professionals in district hospitals Parents of neonates born with gastroschisis	Paediatric and general surgeons Surgical residents, house officers/interns, medical students, medical officers Neonatologists, paediatricians, trainees Nursing supervisor, nurses Gastroenterologists, anaesthetists, dieticians Hospital management Parents of neonates born with gastroschisis

### Patient population

***Inclusion criteria.*** all neonates presenting primarily to the study centre with simple gastroschisis regardless of weight, gestational age or co-morbidities. Neonates with simple gastroschisis do not require surgical intervention and have the potential to be successfully managed with cotside reduction using a preformed silo and sutureless closure without the need for an anaesthetic.

***Exclusion criteria.*** all neonates with ‘complex gastroschisis’ requiring surgical intervention for bowel necrosis, perforation, atresia or other reason.

### Sample size

It is estimated that between 240 to 384 patients with gastroschisis will present to the seven study centres during the 2-year data collection period ( Table 4). An estimated 75% of the neonates with gastroschisis should fulfil the inclusion criteria within the context of the study sites, equating to between 180 and 288 patients in the study in total (67–109 pre-intervention and 113–179 post-intervention). A successful pre-hospital component of the study may result in more patients presenting with simple gastroschisis and hence more being eligible for study inclusion.

**Table 4.** The estimated number of gastroschisis cases per month at each of the study centres.

Site	Estimated no. cases/month	Dates for pre- intervention data collection at each site (total no. months)	Minimum no. pre-intervention cases,	Maximum no. pre- intervention cases	Timeframe for intervention implementation at each site in 2019	Dates for post- intervention data collection at each site (total no. months) *	Minimum no. of post- intervention cases	Maximum no. of post- intervention cases
**Korle-Bu TH,** **Ghana**	1	Oct 2018–May 2019 (7 months)	7	7	May 13 ^th^ – June 7th	May 2019 – Oct 2020 (17 months)	17	17
**Tamale TH, Ghana**	1–2	Oct 2018–June 2019 (8 months)	8	16	June 10 ^th^ – July 5th	June 2019 – Oct 2020 (16 months)	16	32
**Komfo Anokye TH,** **Ghana**	1	Nov 2018–July 2019 (8 months)	8	8	July 8 ^th^ – Aug 2nd	July 2019 – Nov 2020 (16 months)	16	16
**University TH** **Lusaka Zambia**	2–4	Nov 2018–August 2019 (9 months)	18	36	August 12 ^th^ – Sept 6th	Aug 2019 – Nov 2020 (15 months)	30	60
**ADCH, Zambia**	1	Jan 2019–Sept 2019 (8 months)	8	8	September 16 ^th^ – Oct 11th	Sept 2019 – Jan 2021 (16 months)	16	16
**KCH, Malawi**	2–4	Dec 2018–Oct 2019 (10 months)	20	40	October 21 ^th^ – Nov 15th	Oct 2019 – Dec 2020 (14 months)	28	56
**MNH, Tanzania**	2–3	Jan 2019–Nov 2019 (10 months)	20	30	Nov 25 ^th^ – Dec 20 ^th^	Nov 2019 – Jan 2021 (14 months)	28	42
**TOTAL**	**10 – 16**	**60 months**	**89**	**145**	**8 months**	**108 months**	**151**	**239**
**No. of patients** **with simple** **gastroschisis**	**8–12**		**67**	**109**			**113**	**179**

*Each site will collect 24 months of data in total

In this study a convenience sample has been used and hence a post-hoc power calculation has been undertaken. Utilising a two-sided Z-test it has been calculated that at 90% power and p=0.05, the study could detect a difference in mortality of 16% (95% mortality down to 79%) if the minimum number of patients were included and a difference of 12% (95% mortality down to 83%) with the maximum estimated number.

This indicates that the study is appropriately powered. Some centres within sub-Saharan Africa have achieved a mortality rate of 80% or less, suggesting it is an achievable target.

## Stages of implementation

This will involve four key stages: 1) exploration, 2) preparation, 3) implementation, 4) sustainment ^126^.

### 1) Exploration stage

***Context.*** A good understanding and consideration of the local context is vital for the success of the project ^127^. Preliminary data has been collected from study team members through online written correspondence, video meetings and a literature review, as summarised in the Introduction above ^79– 105^. Identified key facilitators and barriers for study success are summarised below and will be further investigated through the systematic review, qualitative study, Delphi process and local in-person co-development meetings. In addition, an institutional capacity assessment will be undertaken at each study site to determine the resources currently available for gastroschisis management ( Supplementary File 3) ^110^. The interventional bundle and implementation strategy will be adapted to overcome such barriers and optimise facilitators.

Summary of key facilitators:

- Local champions with a strong desire to improve outcomes.
- Self-selected teams who have requested to participate in the study.
- The availability of a low-technology, cost-effective interventional bundle with proven effectiveness.
- One to four cases per month at the study sites with a current mortality of over 90–95% and hence the potential to significantly improve outcomes.

Summary of key barriers:

- Staff shortages, particularly nursing staff.
- Staff culture and current beliefs regarding the futility of neonates with gastroschisis ^2, 6^.
- Lack of infrastructure for the provision of neonatal PN and resources for central IV access.
- Problems with infection control and sepsis.

Key strategies to overcome barriers:

- Involvement of all key members of the MDT in the development of the interventional bundle to ensure it is acceptable and feasible within the local contexts.
- Utilisation of maternal input for monitoring, basic care and identification of red flags to help overcome nursing shortages, in centres where it is deemed appropriate.
- Empowerment of nurses through training, resource provision and inclusion in all aspects of the study development, implementation and follow-up. This will include group and one-on-one training and in-practice support from a surgical nurse specialist with experience managing neonates with gastroschisis.
- MDT simulation training to optimise teamwork, networks and solidarity around a shared goal to improve survival in neonates with gastroschisis.
- Input from a paediatric gastroenterologist specialised in PN to identify and trial potential solutions to sourcing and administration of PN for neonates at the study sites. Lessons will be learned from experts managing to administer PN successfully in low-resource environments from the qualitative study analysis.
- Implementation of a standardised protocol of care with proven infection control measures that have been successful in LMIC QI projects.

***Pre-intervention data collection.*** This will determine the current management strategies and the pre-intervention outcomes, both of which are vital to measure and evaluate change. Ineffective or potentially harmful strategies identified through evaluation of the pre-intervention data can be discussed amongst the MDT team and targeted for improvements during the intervention phase.

### 2) Preparation stage

**Figure 1.**
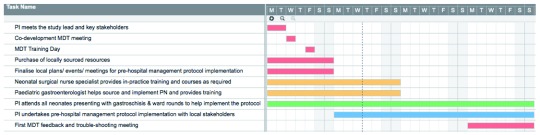
In-country implementation timeline (4-week period).

***In-country co-development meeting.*** The preparation and implementation stages are outlined in Figure 1. Upon arrival at each study site, the PI will meet with the study lead and key stakeholders. A co-development meeting will be held within the first week with all members of the MDT caring for neonates with gastroschisis and other key stakeholders such as hospital management. The study details will be presented, discussed and debated. Adaptations will be made according to the local context as advised by the team. Required resources will be sourced and purchased ( Supplementary File 4) ^110^. A plan will be made with the local team regarding equipment storage for safe keeping and easy access.

***Training***. A training timetable will be devised. Initially, there will be a training day for all MDT members which will include:

1) Initial resuscitation, silo application, reduction and closure using a gastroschisis simulation model.
2) MDT simulation training where all team members practice managing a neonate with gastroschisis in real time from the point of arrival at hospital to silo application and stabilisation.
3) Daily ward management and care (with a nurse training session led by a surgical neonatal nurse specialist with experience managing neonates with gastroschisis).
4) Early breastfeeding and enhanced enteral feeding programme.
5) Infection control and central line management.
6) Parenteral nutrition prescribing, preparation, safe administration and storage (if it is going to be included in the interventional bundle at that site).
7) Parental involvement in neonatal monitoring and care.
8) Patient consent.

The trainers will include the principal investigator (paediatric surgery registrar with experience managing neonates with gastroschisis and use of a preformed silo for reduction and sutureless closure), a neonatal nurse specialist (with experience managing neonates with gastroschisis and infection control), and a paediatric gastroenterologist (specialised in enhanced enteral feeding programmes and parenteral nutrition). The course will be designed by these three healthcare professionals with input from the study leads and lead investigators. Teaching methods will include a combination of didactic and interactive presentations and group simulation training. The latter will utilise a gastroschisis simulation model along with the basic equipment required to resuscitate a neonate with gastroschisis to replicate the real-life situation as much as possible. There will be a pre- and post-training evaluation to identify further training needs that can be undertaken during the 4-week implementation phase (this will not be an assessment, rather a strategy to optimise protocol uptake and fidelity).

***Pre-hospital component.*** A further meeting with the study lead and appropriate stakeholders will be undertaken to confirm details of the pre-hospital component. Key stakeholders from the gastroschisis referral centres surrounding the study centre will be identified and a context-specific implementation strategy developed to implement the pre-hospital management protocol.

### 3) Implementation stage

***Further training and support.*** The PI will spend a total of 4 weeks at each site. During this time the PI will attend every admission and in-hospital delivery of a neonate with gastroschisis to assist the local team with implementation and use of the study protocol. This will include an estimated one to four neonates at each site according to current presentation rates. The PI will attend daily neonatal ward rounds, provide input on patients with gastroschisis on the ward on a daily basis, attend twice-daily silo reductions and be present during ward closures.

A surgical nurse specialist will spend two weeks at each study site, initially to help with the MDT training day and then to spend time on the wards with the nurses in order to assist with in-practice, real time training. Additional training sessions can be planned during this time as required. The aim will be to empower the nurses through resource allocation, training and encouragement from a nurse who regularly successfully manages neonates with gastroschisis.

A paediatric gastroenterologist will spend two weeks at each site to assist with the MDT training day, work with the local team to develop the infrastructure and skills required to provide PN (if included in the protocol at that site) and to provide training on an enhanced enteral feeding programme.

***Pre-hospital component.*** This will be undertaken by the PI and key stakeholders during the 4 week in-country period.

### 4) Sustainment stage

***Monthly MDT feedback and troubleshooting meetings.*** The initial meeting will happen within two weeks of managing the first neonate with gastroschisis following implementation of the protocol and then at monthly intervals thereafter for the duration of the study. When in-country the PI will attend the meeting personally; following that the PI will attend remotely via teleconference. At the meeting a real time update on outcomes will be fed back to the MDT, both the patient outcomes and compliance with the protocol (fidelity). There will be the opportunity to trouble-shoot and decide upon potential solutions to problems encountered. Meetings will be postponed to the following month if no neonates with gastroschisis were managed at the study centre during the preceding month.

## Data collection

### Clinical outcomes

***Primary outcome.***

1) All-cause, in-hospital mortality.

***Secondary outcomes.***

1)   Major complication within 30-days of primary intervention ^1^: secondary bowel ischaemia, necrosis or perforation requiring resection ^2^, abdominal compartment syndrome ^3^ or need for further unplanned surgical intervention.

2)   Length of hospital stay amongst survivors (days).

3)   Time to full enteral feeds (days).

4)   Need for ventilation (yes/ no).

5)   Duration of ventilation (within 30 days of primary intervention in days).

Patient data will be collected on patient demographics, antenatal care, pre-hospital care, clinical condition on arrival, resuscitation, intervention (for bowel reduction and closure), ward care and outcomes ( Supplementary File 5) ^110^.

***Study impact on neonates with other congenital anomalies.*** The all-cause in-hospital mortality pre- and post- intervention will be determined for neonates presenting for the first time with anorectal malformation and intestinal atresia to see if there are indirect benefits for other patients requiring a similar package of neonatal surgical care.

### Service delivery outcomes

1)   Time from birth to arrival at the study centre (hours).

2)   Proportion of patients with clear plastic bowel coverage at the time of arrival to the study centre.

3)   Proportion of patients with intravenous fluids prior to arrival at the study centre.

4)   Time from arrival to primary intervention (hours).

5)   Number and position of MDT members present during the initial resuscitation, primary intervention and defect closure.

6)   Proportion of patients receiving PN at some point during their hospital stay.

All patient data will be collected prospectively using REDCap Data Capture Software ^128^. Data collection forms will be printed for real-time data collection at the patient’s bedside. This can be uploaded later to REDCap. Patient data entered into REDCap will be anonymous and local study teams will maintain a spreadsheet to keep track of REDCap IDs alongside the patient identifiable information. A pilot study was undertaken to ensure the data collection form is usable, appropriately worded and contains all the relevant content prior to project launch.

### Implementation outcomes

***Acceptability, adoption, appropriateness and feasibility.*** These will be evaluated and addressed during the qualitative studies, Delphi process and in-country co-development meeting to optimise the interventional bundle prior to implementation. Definitions are documented in the introduction section above. In order to evaluate these outcomes post-intervention, MDT members and parents will be invited to undertake a validated 12-item survey on acceptability, appropriateness and feasibility during the 4 ^th^ week of the in-country implementation phase and again at the end of the study ( Supplementary File 6) ^110, 129^.

***Fidelity.*** The proportion of management protocol components completed as intended will be assessed using a checklist at the time of preformed silo application and defect closure. The checklist will be completed by the person undertaking the intervention for every neonate included in the study. A second observer, who has been trained in the gastroschisis management protocol, will independently complete the checklist for 50% of the cases.

Fidelity of the implementation process will also be evaluated as follows:

1)   Number of centres where the study leads inputted into the study design and protocol either via video conference with the PI and/or via internet communications.

2)   Total number and position of the MDT members* engaged in the qualitative study and/ or Delphi process from each study site.

3)   Did an in-person co-development meeting take place at the start of the implementation phase at each study site (yes/no).

   If yes, were adaptations made to the interventional bundle accordingly (yes/no).

   If yes, how many of each of the MDT members ^4^ were present during the meeting.

1)   Total number and position of the MDT members completing the MDT training day.

2)   Proportion of referral hospitals ^5^ (as identified by the study leads at each centre) receiving implementation of the pre-hospital management protocol through in-person education and training.

In order to accurately evaluate fidelity of both the interventional bundle and implementation process it will be important to distinguish between non-compliance and purposive adaptations ^130, 131^. Stirman *et al*. have produced a framework and coding system for adaptations of evidence-based interventions that will be used to accurately document any deviations from the original plan at each site throughout the duration of the study ^132^.

***Coverage.*** The proportion of eligible patients who actually receive the intervention will be determined through the data entered into REDCap on all patients presenting with gastroschisis. Neonates with simple gastroschisis included within the study will have all data points completed. Neonates with complex gastroschisis excluded from the study intervention will have baseline data collected on the following: patient demographics, pre-hospital care and outcomes.

***Cost.*** The average cost per patient with gastroschisis will be estimated at the study centres, pre- and post-intervention. The implementation costs will also be calculated. The number of disability-adjusted life years (DALYs) averted through implementation of the interventional bundle will be calculated. The previously utilised disability-weight of 0.2 will be used for surviving neonates with gastroschisis where 0 is no disability and 1 is death ^2^. The cost in US$ per DALY averted will be calculated.

***Sustainability.*** The current intervention and implementation strategy have been evaluated for potential sustainability using the NHS Quality Improvement Sustainability Model ^133^. It scored 69.5/100 (19.8/31.3 for process, 43.1/52.4 for staff and 6.6/16.5 for organisation). A score above 55 is deemed ‘reason for optimism’. This evaluation has highlighted the need to set up a monitoring process beyond the life of the study, involve organisation leaders throughout the study process, to align the project with the team and organisation’s other strategy aims for improvement and to ensure facilities and equipment utilised are sustainable long-term. A re-evaluation will be undertaken just prior to interventional implementation and again following completion of the study.

## Data analysis

### Clinical outcomes

***Pre-post study analysis.*** Primary and secondary clinical outcomes will be compared pre- and post-intervention using chi-squared analysis for categorical variables or Fisher’s exact test if either of the groups contains less than 10 patients. For normally distributed continuous variables an unpaired t-test will be used and for non-normally distributed continuous variables a Mann-Whitney *U* test will be used. P<0.05 will be deemed significant. Cases presenting during the 1-month implementation phase will be deemed post-intervention since the PI will be in attendance at all cases alongside the local team to assist with implementation of the protocol and training will have commenced.

***Time series analysis.*** Time series analysis will be undertaken using Statistical Process Control to distinguish significant trends and shifts in mortality from background variation during the study period ^134^.

***Multivariate analysis.*** Multivariate logistic regression analysis will be used to identify factors affecting all-cause in-hospital mortality with adjustment for confounding factors. Potential confounders include gestational age, weight, presence of co-morbidities, and ASA score at the time of primary intervention.

### Service delivery and implementation outcomes

Regression analysis will be undertaken to evaluate the impact of the service delivery outcomes and implementation outcomes including the acceptability (AIM) score, appropriateness (IAM) score, feasibility (FIM) score and fidelity on all-cause in-hospital mortality.

In order to assess sustainability during the post-implementation phase, fidelity of the interventional bundle will be analysed using time series analysis. This will assess whether fidelity remains stable, increases or declines following implementation. Time-series analysis will also be used to assess all-cause in-hospital mortality during the post-implementation phase to determine if there is an upwards, stable or downwards trend following implementation.

## Data management

Anonymous, de-identified patient data will be entered into REDCap by the study teams. Study teams will be able to access their own patient data, but not data from other study centres. The data will be pseudo-anonymised at a local study centre level – study leads will maintain a separate, confidential list of REDCap codes with patient identifiers to permit patient follow-up and later identification if required. The principal investigator will have access to the full anonymous, de-identified dataset and other team members on the steering committee, expert advisory committee and study steering committee will have access on a need to know basis for data management and analysis purposes. At no stage will the principal investigator or any team members outside of the local study centre have access to the key to the pseudo-anonymised data. Data on REDCap is backed-up on the King’s College London secure server on a daily basis and is managed by King’s REDCap Administration Team. The principal investigator will maintain a weekly back-up of the data on two password protected, encrypted memory sticks. A data management plan has been registered and approved by King’s Data Protection Regulation team.

## Ethical considerations

Ethical approval has been gained at all participating sites. Ethical approval reference numbers: King’s College London, HR-17/18-7107; Korle-Bu Teaching Hospital, KBTH-IRB/00037/2018; Tamale Teaching Hospital, TTHERC/19/06/18/04; Komfo Anokye Teaching Hospital, CHRPE/AP/616/18; University Teaching Hospital Lusaka, 063-08-18; Arthur Davison Children’s Hospital, TRC/C4/01/2019; Kamuzu Central Hospital, P.05/18/2398; Muhimbili National Hospital, NIMR/HQ/R.8a/Vol.IX/2844.

### Patient consent

Written consent will be required from the guardian holding parental responsibility for patients included in the study ( Supplementary File 7) ^110^. Parents will be provided with an information leaflet in their own language and they will have a detailed discussion with a member of the study team to ensure they understand the potential benefits, risks and alternatives to participating in the study ( Supplementary File 7) ^110^. All parents will be informed that there is no obligation to participate and if they do agree to participate, they are free to withdraw at any stage. Patient’s legal guardians will be able to request access to their own child’s data. At the time of consent for participation in the study, consent will also be sought to openly publish their child’s anonymised data. All study team members will receive training on parental consent for research during the MDT Training Day. If any ethical issues arise during the project, they will be discussed amongst the lead investigators and study site leads, the relevant ethical committees and the study steering committee.

### Study Steering Committee (SSC)

An SSC consisting of two academic paediatric surgery consultants will independently oversee the project to ensure it is ethically sound throughout ( Supplementary File 8) ^110^. They will have access to the anonymous patient data collected in real time. If any major complications or deaths occur that may be associated with the intervention, these will be discussed and addressed between the SSC and study teams.

It is unlikely that the interventional care bundle will worsen outcomes compared to the current situation of above 95% mortality across the study centres. The main potential risk is bowel ischaemia related to incorrect application of the silo from either torsion of the vessels, use of a silo that is too small or an abdominal wall defect that is too small requiring incision under local anaesthesia ^135^. Training will be provided to avoid this complication and to recognise the early signs of bowel compromise, with techniques to remedy the situation before irreversible ischaemia and necrosis occurs. Of note, studies have shown bowel ischaemia is just as common in neonates receiving primary closure; however, in the latter, the problem cannot be visualised and remedied as easily ^54, 55^. Overall, use of a preformed silo is less invasive than the alternative of surgical interventional and general anaesthesia and hence carries less risks to the patient, especially in a low-resource environment without the availability of neonatal intensive care facilities. Insurance will be provided through King’s College London for any harm caused to patients receiving the care in the study protocol.

### Unintended consequences

Potential unintended or indirect consequences of the study, both positive and negative, must be considered.

Positive consequences may include:

•   Improved team building/ interaction/ communication with benefits for a wider range of patients.

•   Improved generic neonatal care and resuscitation skills and infection control awareness with potential benefits for a wider range of patients.

•   Development of infrastructure and supply routes required to enable delivery of neonatal PN with benefits to a wider range of patients.

•   Enhanced research capacity.

•   Enhanced CV’s and career progression amongst those involved.

•   Greater staff job satisfaction with the potential for improved retention rates.

•   Development of networks with international partners with the potential for future collaborative projects.

Negative consequences may include:

•   Potentially less time with other patients.

•   Disruption of study team members work/life balance.

•   Risk of reduced survival/increased complications in those few (5-10%) with simple gastroschisis who may have survived with primary closure in theatre if available.

•   Risk of prolonged hospital stays and resource utilisation without improved survival.

The above possible negative consequences will be discussed with study team members and a plan instigated to minimise risk. Parents will be informed both in writing and verbally of both the potential benefits and risks associated with participation in the study at the time of consent.

## Discussion

### Study limitations

•   Active involvement of the study team members in the development of the interventional bundle could result in changes being made prior to implementation of the interventional bundle. This could result in a lack of significant difference pre- and post-intervention.

•   Longer term follow-up would be ideal to assess for sustainability after study completion.

•   The in-country implementation phase is limited to 4-weeks at each site. Hence, only 1–4 patients with gastroschisis will be expected to present during this time in order to utilise the new management bundle whilst the PI and team are present. However, MDT simulation training using a gastroschisis model will permit additional hands-on training and troubleshooting.

### Dissemination of results

All study team members will be involved in the dissemination of results through local, regional, national and international conferences. The results will be submitted for open-access peer-reviewed publication in a high impact journal. Study team members who have contributed to the design, undertaking, analysis and write-up of the study will be included as authors. Other teams members who have contributed to the study, but do not fulfil authorship criteria, will be acknowledged. Following publication, the full anonymised dataset will be made openly available to the public. Where all necessary approvals have been obtained, qualitative data will be converted into a suitable format for public dissemination and deposited in the open access UK Economic and Social Data Service (ESDS).

### Study impact

The systematic review, qualitative study and Delphi process will all provide unique evidence towards optimising care for neonates with gastroschisis in low-resource settings. Each study will be submitted for publication. The interventional study will allow for that evidence to be tested in-practice across seven tertiary paediatric surgery centres in sub-Saharan Africa. The study design will enable detailed analysis of which components of the interventional bundle and implementation strategy were and were not effective. Including multiple sites in the study will determine the generalisability of the intervention and will permit a more detailed analysis of contextual factors affecting outcome.

If successful, funding will be sought to scale-up the intervention to other sites across sub-Saharan Africa and then LMICs globally using a stepped-wedge approach. This has the potential to save the lives of hundreds of neonates born with gastroschisis. Since the majority of neonates with gastroschisis typically go on to live a full and normal life, the number of DALYs averted is very high. Gastroschisis has been described as a ‘bellwether procedure’ for neonatal surgery in low-resource settings ^2^. Hence, potentially improving the care for neonates with gastroschisis could also improve outcomes for neonates with other congenital anomalies that require a similar package of neonatal surgical care. This could have a wider global health impact in light of the recent Global Burden of Disease Report findings that congenital anomalies have recently risen to become the fifth-leading cause of death in children under 5 years globally ^136^.

## Data availability

### Underlying data

All data underlying the results are available as part of the article and no additional source data are required.

### Extended data

Open Science Framework. Study Protocol: Developing and implementing an interventional bundle to reduce mortality from gastroschisis in low-resource settings. https://doi.org/10.17605/OSF.IO/M9DKB ^110^.

The following extended data are available:

- Supplementary File 1: Driver diagram summarising the primary and secondary drivers to achieving improved survival of neonates with gastroschisis in sub-Saharan Africa.
- Supplementary File 2: NoMAD validated survey for evaluating the four constructs of the Normalisation Process Theory in practice.
- Supplementary File 3: Institutional Capacity Assessment for Gastroschisis Management.
- Supplementary File 4: Study resources and justification
- Supplementary File 5: Gastroschisis Interventional Study Data Collection Form.
- Supplementary File 6: Validated surveys to evaluate implementation outcomes: Acceptability of Intervention (AIM), Intervention Appropriateness Measure (IAM) and Feasibility of Intervention Measure (FIM).
- Supplementary File 7: Parent information leaflet and consent form.
- Supplementary File 8: Study Team.

### Reporting guidelines

Open Science Framework: SPIRIT checklist for “Developing and implementing an interventional bundle to reduce mortality from gastroschisis in low-resource settings”. https://doi.org/10.17605/OSF.IO/M9DKB ^110^.

Data are available under the terms of the Creative Commons Zero "No rights reserved" data waiver (CC0 1.0 Public domain dedication).

## Footnotes

^1^Defined as the first bowel intervention the neonate received including silo application or primary closure.

^2^Defined as bowel necrosis or perforation that was not present prior to primary intervention and hence occurred after primary intervention.

^3^Defined as respiratory insufficiency secondary to compromised tidal volumes, decreased urine output by falling renal perfusion (<1ml/kg/hr) and any other organ dysfunction caused by increased intra-abdominal pressure (based on clinical judgement – the pressure does not have to be measured to fulfil the criteria).

^4^MDT members include ward nurse, study lead nurse, neonatology consultant, neonatal resident, paediatric consultant, paediatric resident, paediatric surgeon, general surgeon, surgical resident, intern/house officer, non-physician assistant, doctor anaesthetist, nurse anaesthetist, hospital management, other.

^5^Referral hospitals will be defined as those sites that referred patients with gastroschisis during the pre-implementation data collection or sites known to the study lead to have referred two or more patients with gastroschisis within the 2-years prior to the study
