# Supplementary material for: Developing and implementing an interventional bundle to reduce mortality from gastroschisis in low-resource settings
Source: Wellcome Open Res. 2019 Mar 8;4:46. [Version 1] doi: 10.12688/wellcomeopenres.15113.1 (PMC6456836; doi:10.12688/wellcomeopenres.15113.1)
Supplement: Peer review history

# Peer review: Reviewer response for version 1

**DOI:** 10.21956/wellcomeopenres.16489.r35046

**Published:** 2019-04-08

**Authors:** Nick Lansdale

**Affiliations:** 1 Department of Paediatric and Neonatal Surgery, Royal Manchester Children's Hospital, Manchester, UK

This protocol aims firstly to develop and then implement and evaluate an interventional bundle to improve outcomes of gastroschisis in LMICs. This is a commendable initiative and one that has been well considered by a good team of researchers. The continued high mortality from gastroschisis in LMICs and the large disparity with HICs makes this a good target for intervention, with strong potential for significant improvement.

 The mixed methods study design (qualitative study followed by a Delphi process and then quantitative analysis of outcomes post implementation) is appropriate and stands a good chance of success if appropriate resource is given to all of these aspects. Given that each later step depends on success of the previous step, it is important that all are completed satisfactorily and in a timely fashion.

 The implementation phase and subsequent evaluation are described in detail and are well designed to meet the relevant study objectives. The team appear to have good expertise in these methodologies and this element of the study is a real strength. The planned data analysis is appropriate. In the discussion section the potential limitations of the study are acknowledged and a strategy for tackling potential ‘recruitment’ problems is outlined. However, there does appear to be a genuine risk of the relatively short (4 week) centre visits being compromised by there not being any cases of gastroschisis during the stay: simulation training may help this but the effectiveness of the visit will almost certainly be compromised by a lack of cases. I wonder whether longer stays were considered, potentially covering fewer centres?

 My principal concerns relate to the development phase for the interventional bundle and somewhat echo Peter Saula’s review. Firstly, I’m a little unsure as to exactly how much additional information will be gained from a new systematic review, if it only considers interventions to reduce mortality from gastroschisis in LMICs (there is unlikely to be much published literature here and the study team will no doubt be aware of most of it already). There is also no real detail of the systematic review methodology.

 Secondly, whilst it is stated that protocols are ‘forthcoming’ for the qualitative study and Delphi process, if these study elements are to be included in this protocol (and progress to bundle implementation is dependent on their successful completion), then more details are essential. Currently it is unclear as to what these elements will entail: if they are to robustly influence the intervention (as they must do to gain maximum impact from them), then they need to be awarded the same level of importance as the implementation and evaluation phase. This is particularly true with regard to the amount of time allocated to them: I was unable to find any indication of this in the protocol but I would have thought a high quality qualitative study that incorporated clinician interviews (with recording, transcription and thematic analysis etc.) and was followed by a 2 or 3 stage Delphi process, could take more than a year to complete. This would mean the final design of the bundle and then implementation could not occur for quite some time. I presume this is not the case though, as the dates of the centre visits start in May 2019: does this mean the systematic review, qualitative phase and Delphi have already been done or are they not seen as essential prior to implementation? I think this needs clarifying and in an ideal world I believe these initial ‘development’ phases should have been completed first.

 Overall, this promises to be a very good study with real potential for improving outcomes: I wonder whether it is perhaps a little over ambitious and (entirely understandably) trying to accomplish too much in a relatively short time frame.

I have read this submission. I believe that I have an appropriate level of expertise to confirm that it is of an acceptable scientific standard, however I have significant reservations, as outlined above.

# Peer review: Reviewer response for version 1

**DOI:** 10.21956/wellcomeopenres.16489.r35044

**Published:** 2019-03-27

**Authors:** Cristian Zalles-Vidal

**Affiliations:** 1 Department of Pediatric Surgey, Hospital Infantil de México Federico Gómez (HIMFG), Mexico City, Mexico

This protocol is a great effort to reduce mortality by gastroschisis in seven tertiary centers in Africa. All the phases of the study are well described, and it analyzes the normalization of the process. 

 I consider that in the development of the Delphy process the participation of members from the seven centers in Africa is important, because they know the reality of the hospital and the patients that are going to be treated. Therefore, this bundle can work well in this environment but could not have the same result in some other. This difference between the hospitals and the patients can modify the result, but the authors found a good solution using the adaptable components of the bundle to control it. In the Stakeholders part of the protocol it is mentioned that the study leads at each center will be invited to participate in the Delphy process, but in the Delphy process segment they mention that “if required”  a consensus will be made.

 Does the order to begin the study in the different centers have a reason? Because I think it would be better if you start with the one showing more cases per month; in this way, the team that is going to teach how to apply the bundle will have a bigger exposure to the process of learning of the MDT.

 The service delivery outcomes are going to show if the changes in the management are being used, but I believe it is important to measure if the intervention has a good result. In point number 2 it would be good to know the condition of the bowel (matting) at arrival to the hospital, sometimes if the plastic coverage leaks the bowel dries and forms an important matting that results in difficulties during later treatments. In point number 3, it would be good to know if the patient presents a good hydro electrolytic state at arrival (normal urinary output).

 Have you considered an Orogastric tube for the pre hospital components? This could help the bowel reduction, especially if the patient takes several hours to arrive to the hospital were the specialized treatment will be established.

 I think intestinal atresia is difficult to diagnose in some cases of gastroschisis, especially if the bowel has an important peel. Maybe some considerations should be done when the team is not completely sure of the atresia, the patient should not be excluded from the study.

 One limitation of the study is that in the in-country implementation phase only 1 to 4 patients will be treated, simulation is a good tool to improve the adherence to the bundle. Have you considered the use of video of the subsequent cases to review the technique of the silo placement and the sutureless closure? A guided analysis of the real cases by all the MDT could be a good way to share your experiences.

I have read this submission. I believe that I have an appropriate level of expertise to confirm that it is of an acceptable scientific standard.

# Peer review: Reviewer response for version 1

**DOI:** 10.21956/wellcomeopenres.16489.r35047

**Published:** 2019-03-25

**Authors:** Peter Saula

**Affiliations:** 1 School of Medicine, Moi University, Eldoret, Kenya

This project is well conceptualized, relevant and has a potential to make a great impact in the management and outcomes of gastroschisis in Sub-Saharan Africa/other low-resource settings.

 The rationale of the study is clearly discussed in the introduction and the literature review. The study aim and objectives are well designed, clear and achievable.

 The definitions of terms and concepts are clear and precise.

 A few areas of concern include:

**Methods:**

 The hybrid Type 2 effectiveness-implementation pre-post study design is appropriate to achieve the objectives of the study. This basically involves both quantitative and qualitative methods that would clearly augment each other.

 However, the description of the study population is vague. The development of the gastroschisis interventional bundle as described in the protocol will involve - systematic review of literature; analysis of qualitative data collected by semi-structured interviews of neonatal surgical care providers in LMICs and the Delphi process; and analysis of quantitative data of the clinical outcomes of gastroschisis before and after the implementation of the gastroschisis interventional bundle. In order to enrich this protocol, each of the above mentioned aspects of the project methodology may be considered separately as "mini-" studies that warrant clear description of the study population, sample-size determination, sampling procedure, inclusion-exclusion criteria, and clearly defined study procedures. As it is now, emphasis has been laid on the quantitative aspect with no clear description of how the other aspects will be done.

 Other than the neonates with gastroschisis who meet the inclusion criteria, the study population ought to include neonatal surgical care providers and the parents/guardians who will for sure provide analyzable research data in the qualitative aspect of this study.

 On the section on data collection-clinical outcomes, neonates with other congenital anomalies - ano-rectal malformations & intestinal atresia - also seem to creep into your study population, why is this so? What would this do to the internal validity of your study?

 What about the inventory of the available resources for managing neonates with gastroschisis in the selected tertiary care centres? Would you include this in the study population?

**Exclusion criteria:** All neonates with "complex gastroschisis", defined as the presence of bowel necrosis, perforation and intestinal atresia, will be excluded in the study. How will the investigators particularly isolate those with intestinal atresia, given its prompt diagnostic challenge?

**Sample Size determination:** The described statistical calculation of the sample size powers the quantitative aspect of the study, what about the systematic literature review and the qualitative aspect? The authors need to clearly describe the sampling methods for each aspect of this study.

**Data analysis strategy: **It is well designed for the quantitative aspect of this study, however, the data analysis strategy for the systematic literature review, the qualitative aspect as well the Delphis process ought to be described concisely.

**Ethical Considerations:** This section largely ignores the other aspects of the study. The consenting process described is for a section of study participants namely the neonates. However, this study is deemed to collect analyzable research data - quantitative & qualitative - from many more study participants who may include the neonatal surgical care providers, parents/guardians and perhaps the management staff of the selected tertiary level hospitals. I suggest that the consenting process be broadened to include all the probable study participants.

**Overall Comment: **A very well written study protocol that targets an area of surgical care with paucity of scientific literature, yet in dire need of standardized care protocols.

I have read this submission. I believe that I have an appropriate level of expertise to confirm that it is of an acceptable scientific standard, however I have significant reservations, as outlined above.
